# An evaluation of the effects of intensity and duration on outcomes across treatment domains for children with autism spectrum disorder

**DOI:** 10.1038/tp.2017.207

**Published:** 2017-09-19

**Authors:** E Linstead, D R Dixon, E Hong, C O Burns, R French, M N Novack, D Granpeesheh

**Affiliations:** 1 Machine Learning and Assistive Technology Laboratory, Schmid College of Science and Technology, Chapman University, Orange, CA, USA; 2 Department of Research and Development, Center for Autism and Related Disorders, Woodland Hills, CA, USA; 3 Department of Psychology, Louisiana State University, Baton Rouge, LA, USA

## Abstract

Applied behavior analysis (ABA) is considered an effective treatment for individuals with autism spectrum disorder (ASD), and many researchers have further investigated factors associated with treatment outcomes. However, few studies have focused on whether treatment intensity and duration have differential influences on separate skills. The aim of the current study was to investigate how treatment intensity and duration impact learning across different treatment domains, including academic, adaptive, cognitive, executive function, language, motor, play, and social. Separate multiple linear regression analyses were used to evaluate these relationships. Participants included 1468 children with ASD, ages 18 months to 12 years old, *M*=7.57 years, s.d.=2.37, who were receiving individualized ABA services. The results indicated that treatment intensity and duration were both significant predictors of mastered learning objectives across all eight treatment domains. The academic and language domains showed the strongest response, with effect sizes of 1.68 and 1.85 for treatment intensity and 4.70 and 9.02 for treatment duration, respectively. These findings are consistent with previous research that total dosage of treatment positively influences outcomes. The current study also expands on extant literature by providing a better understanding of the differential impact that these treatment variables have across various treatment domains.

## Introduction

Interventions based on the principles of applied behavior analysis (ABA) are considered effective for individuals with autism spectrum disorder (ASD).^1, 2, 3, 4^ Although there is strong evidentiary support for the use of ABA, there is evidence of heterogeneity in response to treatment. Researchers tend to approach this heterogeneity by considering predictors of treatment response that are either child specific or treatment specific.^5^ Researchers have examined whether child-specific characteristics, such as age,^6, 7, 8, 9^ autism symptom severity,^10, 11^ cognitive functioning^10^ and adaptive functioning,^8^ impact response to treatment. Treatment-specific variables, such as treatment intensity,^4, 6, 12^ practitioner or teacher training,^13, 14^ treatment location^15^ and clinical supervision,^16^ have also been investigated. The amount of treatment, or treatment ‘dosage,’ is one of the most widely studied aspects of ABA intervention.

Despite findings that long-term comprehensive ABA leads to positive effects in multiple domains of behavior,^4^ there is still debate over the required level of treatment intensity (that is, hours per week) to achieve optimal gains. Granpeesheh and colleagues^6^ found that greater treatment hours and younger age at intake predicted larger gains in the number of mastered objectives. Further, the results of Eldevik and colleagues^17^ indicated that a high treatment intensity (that is, 36 or more hours per week) was the only variable that independently predicted IQ and adaptive gains, which supports previous findings that treatment intensity is a reliable predictor of ABA intervention outcomes.^18, 19^ Recently, Linstead and colleagues^12^ found that increased treatment hours predicted greater progress, with treatment dosage accounting for 60% of the variance in mastered learning objectives when using artificial neural networks. In addition to treatment intensity, other facets of treatment dosage, such as treatment duration, have been explored. Virués-Ortega^20^ investigated treatment duration in addition to intensity, finding greater treatment intensity and longer treatment duration to have positive effects on intellectual functioning, language and adaptive behavior. In a later study, Virués-Ortega *et al.*^9^ found that total dosage (that is, a combination of intensity and duration) was the single predictor of and the highest contributor to treatment outcomes. While total dosage was found to optimize outcomes, effects were not strong enough to report treatment duration alone as an outcome predictor. Overall, the literature indicates that increased treatment intensity has significant positive effects on progress and skill improvement, but the effect of treatment duration requires additional study.

It is difficult to get a clear picture of how these factors impact treatment outcomes due to inconsistency across studies in regards to the measurement of progress or skill improvement. Researchers typically use standardized assessments to measure treatment progress and outcome,^21^ whereas clinicians typically rely on the number of mastered learning objectives,^22^ which is determined based on direct observation. Direct observation of behavior can provide information on a number of different abilities at a particular point in time, whereas standardized assessments provide a broad picture of overall treatment outcome. Granpeesheh and colleagues^6^ suggested that using mastered learning objectives as a dependent variable in treatment studies would allow for the measurement of short-term outcomes, which diagnostic scales are not designed to detect. In addition, mastered learning objectives could serve as an intermediate measure between the day-to-day data collected on particular occurrences of behavior and the more general outcomes measured by standardized assessments. Therefore, this variable has particular social significance as it evaluates whether the specific skills being targeted are mastered by the participant.

The current study aimed to expand on the findings of Virués-Ortega^20^ by evaluating the effect of treatment dosage (that is, intensity and duration) on learning across eight treatment domains found to be relevant to the treatment of ASD: academic, adaptive, cognitive, executive function, language, motor, play and social skills.^23^ Mastered learning objectives in each of these domains were used to measure treatment progress. Investigation across different treatment domains may illuminate whether treatment dosage impacts learning in some domains more than others.

## Materials and methods

### Participants and data collection

Retrospective data from the Skills database were used in the present study. Skills includes a well-validated assessment that evaluates functioning across eight treatment domains^24, 25^ and tracks ongoing treatment progress. Table 1 provides several examples of target lessons and their respective learning objectives, as reported in Skills. Operational data (for example, treatment hours) were collected by the participants’ treatment centers. The data set used in the present study is similar to that described in additional detail by Linstead and colleagues.^12^

To be included in the present study, participants were required to meet the following criteria: were between 18 months and 12 years old, received a minimum of 20 h of ABA treatment per month, and completed at least one full month of treatment. Given that all treatment programs are individualized to target skills based on the participant’s current abilities, the initial intake period often involves probe sessions to identify skills to target during treatment. Therefore, participants who had received less than one month of treatment were excluded, as their data may not be representative of a typical treatment period. These criteria were applied to a pool of 2471 children and resulted in a sample size of 1468 participants. Participants included in the study had a diagnosis of ASD,^26^ autistic disorder,^27^ or pervasive developmental disorder-not otherwise specified (PDD-NOS)^27^ by an independent licensed clinician (for example, psychologist, pediatrician and so on). The mean age of participants was 7.57 years, s.d.=2.37, with a range from 1.83 to 12 years. The average number of hours received per month was 58.80, s.d.=29.54, with a range from 20.08 to 210.36. The mean treatment duration in months was 13.96, s.d.=10.04, with a range from 2 to 36. Of the 1468 included patients, 1156 were male and 312 were female. Participants resided and received behavioral intervention services in the states of Arizona, California, Colorado, Illinois, Louisiana, New York, Texas and Virginia.

### Behavioral intervention services

All participants received behavioral intervention services from a community-based service provider, operating multiple treatment centers. Treatment programs followed the Center for Autism and Related Disorders Model of ASD service delivery^22^ and were customized to address the individual strengths and deficits of the child. Local and regional variables influenced treatment location, which included home, school, clinic or a combination of settings. Clinical recommendations for treatment hours were subject to funding source (for example, insurance, public and so on) determinations, among other variables. Despite the individualization of each participant’s treatment program, these elements were common to all: (a) treatment was delivered on a one-to-one basis by trained behavioral therapists; (b) treatment included both more-structured (that is, discrete trial training) and less-structured (that is, natural environment training) behavioral teaching strategies; (c) language intervention took a verbal behavior approach; (d) both errorless and least-to-most prompting strategies were used; (e) all major empirically validated behavioral principles and procedures were used (that is, reinforcement, extinction, stimulus control, generalization training, chaining and shaping), as appropriate; (f) assessment and treatment of challenging behaviors followed a function-based approach; (g) parents were included in all treatment decisions and received training on a regular basis; (h) direct supervision was provided frequently (for example, biweekly) by an expert in behavioral intervention for children with ASD; and (i) treatment content was based upon the Center for Autism and Related Disorders Curriculum. For a more detailed description of the treatment model, see Granpeesheh and colleagues.^22^

### Data analysis

To examine the impact of treatment intensity and duration on skill acquisition across domains (that is, academic, adaptive, cognitive, executive function, language, motor, play and social), separate regression analyses were conducted. The data set included mastered learning objectives across each domain within a 36-month period (from 1 January 2014 to 31 December 2016). Learning objective mastery criteria were defined as a demonstration of greater than 70% accuracy over at least two treatment sessions conducted across separate days.

Treatment duration was defined as the number of months, within the 36-month period, that the participant received treatment for each treatment domain. A month was counted if at least one learning objective was targeted in a domain. Duration data may not reflect a participant’s total lifetime duration spent on a specific domain. That is, some participants may have been receiving treatment before the 36-month period analyzed in the present study, while other participants may have initiated services during this timeframe.

Unlike the treatment duration data, treatment intensity data required a transformation from its originally recorded state in order to be evaluated across each domain. Total treatment hours for each participant were recorded for all treatment sessions; however, recorded treatment hours were not broken down by subject matter. Using the participant’s overall intensity to evaluate each domain would have been problematic, specifically in the domains that the participant practiced infrequently. If these values were left unadjusted, unnecessary noise and variance would have been introduced when including participants whose treatment did not focus on a particular domain. To remedy these issues, a participant’s total treatment hours for each month were distributed proportionally to the number of learning objectives that were targeted in treatment across each domain relative to the total number of objectives targeted across all domains. Once calculated for each month, the adjusted monthly treatment hours for each domain were averaged to determine the intensity variable. The mean adjusted monthly treatment hours for each domain were as follows: academic, *M*=10.55; adaptive, *M*=5.95; cognitive, *M*=6.38; executive function, *M*=7.13; language, *M*=24.94; motor, *M*=6.01; play, *M*=7.28; social, *M*=11.95.

After preparing the data above, a multiple linear regression model was fit to each domain, with duration and treatment intensity serving as the independent variables, and mastered learning objectives serving as the dependent variable. No additional covariates were included. Regression parameters were estimated via least squares using the R statistical computing environment.^28^

## Results

All eight domains demonstrated a strong linear relationship between skill acquisition and both treatment intensity and duration. The results of the regression models for each domain are shown in Table 2. Each of these models is discussed in further detail, including a summary of the unstandardized regression parameters, which represent the slope of the linear relationship between an independent variable and a predictor. In addition, the *R*-squared statistic, which captures the amount of variance accounted for by the predictors within the underlying multiple linear regression model, is provided below.

**Figure 1:**
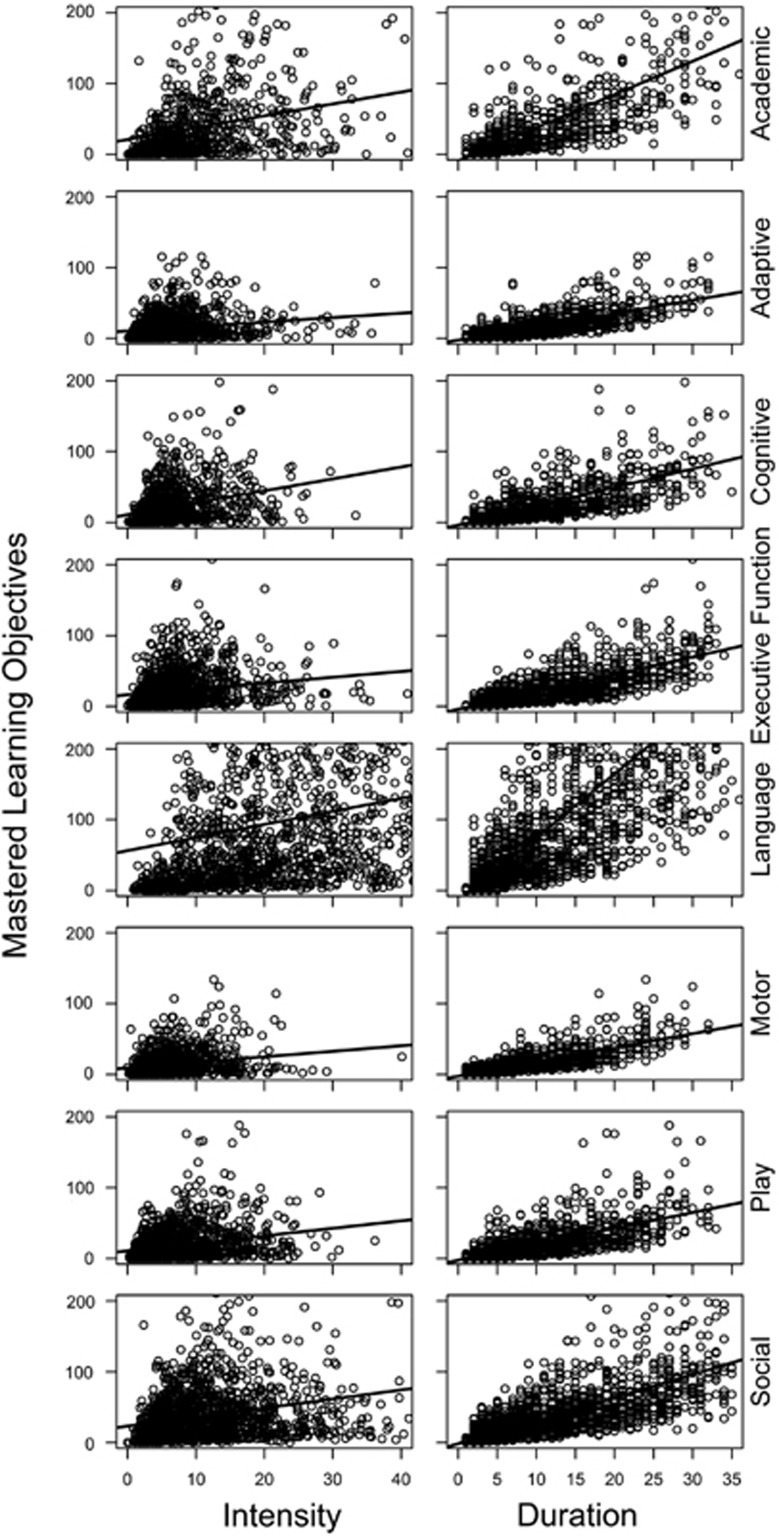
Two-dimensional linear model projections for each treatment domain.

For the academic domain, the coefficients for intensity and duration were 1.68 and 4.70, respectively. See Figure 1 for a two-dimensional scatter plot of the model. Table 2 also demonstrates that the academic domain had the third largest effect size of intensity and the second largest effect size of duration. This model resulted in an *R*-squared value of 62%, which suggests that 62% of the variance in mastered learning objectives within the academic domain can be explained by these two variables alone. For the adaptive domain, the coefficients for the model were 0.63 and 1.88 for treatment intensity and duration, respectively. Despite having the lowest effect sizes for both variables, the *R*-squared value for this model was the second highest of all domains at 65%. The model of the cognitive domain had coefficients of 1.69 for treatment intensity and 2.64 for treatment duration. The *R*-squared value of the cognitive domain model was 59%. The executive function domain had an effect size for treatment intensity of 0.83 and had a coefficient for treatment duration of 2.46. The *R*-squared value for the executive function domain was 60%. The language domain had the highest effect sizes for both treatment intensity and duration at 1.85 and 9.02, respectively. The strong effect of duration was nearly double the effect size of any other domain. The language domain model had an *R*-squared value of 63%. The motor domain had positive effects in intensity and duration with effect sizes of 0.78 and 2.01, respectively. The model showed a strong linear relationship with an *R*-squared value of 67%, which is the highest of all domains. The play domain showed positive linear relationships with coefficients of 1.06 for intensity and 2.25 for duration, with an *R*-squared value of 58%. Finally, the social domain had coefficients of 1.28 and 3.27 for treatment intensity and duration, respectively. The social domain had the lowest *R*-squared value of 50%. The multiple linear regression models for each individual domain were significant, *p*<0.001.

## Discussion

The primary purpose of this study was to evaluate the effect of treatment intensity and duration on the number of learning objectives mastered by children with ASD across eight curricular domains (that is, academic, adaptive, cognitive, executive function, language, motor, play and social). The results of the present study revealed that treatment intensity (for example, hours per week) and treatment duration (for example, months of treatment) had significant effects on all eight domains. Further, the current study found dose–response relationships to be stronger for some domains than for others, with relatively stronger impacts observed in the academic and language domains.

The current study was an expansion on the findings of Virues-Ortega.^20^ The results of the present study are consistent with those reported by Virues-Ortega,^20^ which demonstrated that long-term, intensive ABA intervention produces large, positive effects on language-related outcomes (that is, IQ, receptive and expressive language and communication) and moderate, positive effects on non-verbal IQ, social functioning, and daily living skills in children with ASD. The current study improved on some of the limitations noted by Virues-Ortega,^20^ namely small sample sizes, variability in samples across the three reported domains, and heterogeneity in tools used to measure treatment outcome. The overall sample size of the current study is among the largest published in ASD treatment literature (*N*=1468). Further, all participants in the study were measured according to the same criteria, using a valid, reliable assessment and treatment-tracking tool (that is, Skills).^24, 25^

As reported in other studies,^6, 12^ the current data indicate a positive linear relationship between treatment intensity and the number of mastered learning objectives. That is, an increase in treatment hours predicted a higher number of mastered learning objectives in children with ASD receiving community-based ABA intervention. Although there were positive relationships across all domains, the highest effect sizes were observed in the language, cognitive, and academic domains. Increased treatment duration also predicted a higher number of mastered learning objectives with the greatest impacts observed in the academic and language domains.

These findings have a number of implications. First, these data indicate that both treatment intensity and treatment duration predict mastery of learning objectives. Although the effect of total dosage (that is, interaction between treatment intensity and duration) was not measured in the model, the current study did estimate the percentage of variance in treatment response that was accounted for by treatment intensity and duration together. Treatment intensity and duration alone accounted for a large percentage of the variance found in the mastery of learning objectives within each domain (ranging from 50 to 67%), which supports the findings of Linstead and colleagues^12^ that total dosage accounted for a large percentage of variance in treatment response (*R*^2^=60%). In addition, treatment duration had a stronger impact than intensity on treatment outcomes across all domains. This may suggest that some skills cannot be acquired in a shorter period of time, regardless of the intensity, and that they may require long-term treatment, potentially over developmental periods. Future investigations should further evaluate the effect of treatment duration on outcome. A considerable amount of ABA research has focused on the role of treatment intensity on treatment outcomes,^6, 12, 17, 18, 19^ but there is no empirical evidence indicating that high intensity without consideration for duration will yield optimal results.^29^

Another important implication is that dose–response relationships were stronger for some curricular domains than for others. The current findings provide a fine-grained analysis of treatment dosage relationships across particular treatment domains and further insight into how clinicians can monitor and set treatment expectations. For example, clinicians should pay particular attention to the impact that duration has on the academic and language domains. While treatment intensity had a significant effect on these two domains in the present study, treatment duration had a much stronger relative impact on these outcomes. To achieve greater treatment outcomes, clinicians should target academic and language skills at a high intensity, over a long period of time.

Moreover, the greatest percentages of treatment response variance accounted for by treatment intensity and duration were found in the motor and adaptive domains. This suggests that within these domains individuals tend to respond to treatment intensity and duration in a more uniform fashion (that is, with less influence from other child-specific or treatment-specific variables). While both intensity and duration had a significant effect on treatment response in the motor and adaptive domains, these domains had the lowest effect sizes (that is, learning occurred at a slower rate). Given this, clinicians should expect fewer targets to be mastered overall if the adaptive and motor domains are the sole focuses of treatment. This is consistent with findings by previous researchers who have reported that measures of adaptive behavior tend to have a weak treatment response.^30, 31^

Within the executive function domain, a markedly greater effect size was observed for treatment duration in comparison to treatment intensity. When targeting executive function skills, it may be recommended that clinicians consider applying fewer hours per week (that is, lower intensity) on this domain in favor of targeting these skills over a longer period of time (that is, longer duration). Executive function skills may be slow to change, thus requiring repeated exposure over time.

Interestingly, strong yet balanced effects between treatment response and treatment intensity and duration were observed within the cognitive, play, and social domains, which tend to overlap considerably with the core deficits of ASD. To date, there is a dearth of information evaluating the effects of treatment hours on domains commonly associated with the core symptoms of ASD (that is, deficits in social communication and social interaction, as well as restricted, repetitive patterns of behavior). Most research evaluating the effects of treatment hours relied on standardized assessment measures of IQ or cognitive functioning, language, and adaptive skills.^32, 33, 34, 35^ While overall mastery of learning objectives has also been used to explore the effects of treatment intensity,^6, 12^ it too has limitations when considering change to the core symptoms of ASD.^12^ The current study improves upon these limitations by measuring mastery of exemplars in specific treatment domains; however, it should be noted that the current study did not include evaluations of restricted, repetitive behavior, a diagnostic symptom of ASD. Nevertheless, using mastery of learning objectives across domains as a measure of treatment response is a more representative evaluation of ABA treatment programs and can provide more insight than standardized assessment measures.

It is important to note the variance in the distribution of mastered learning objectives across domains. Figure 1 shows that the language domain had the broadest distribution (that is, more variance in outcomes) of all the curricular domains, specifically for intensity. The source of this variance is not accounted for by the regression model. However, further consideration of the treatment variables does give some suggestions as to likely sources. First, the language domain had the largest number of treatment hours allocated per month (*M*=24.94) to target objectives compared with the other curricular domains. Therefore, the broad distribution of language outcomes may be explained by the way treatment was conducted. That is, language objectives were targeted more frequently compared to other domain objectives. Second, this large variance may also be attributed to the idea that the language domain has a broader spectrum of mastery. On the basis of the child’s current language level, targets within the language lessons may have been broken down into multiple objectives, thus increasing the variance in mastered objectives. The current findings warrant further evaluation of the variance in outcomes across curricular domains.

Another implication is that high treatment dosage yields positive treatment outcomes for children across a wide range of ages (*M*=7.57). This finding challenges the commonly held perception that only young children with ASD may benefit from intensive ABA treatment. Granpeesheh and colleagues^6^ reported that maximizing treatment hours during the younger ages (that is, under 7 years old) may potentially yield greater treatment gains. Nevertheless, without taking into account any child-specific variables including age, treatment intensity and treatment duration alone accounted for a significant portion of treatment progress across all curricular domains. These findings also have implications for expectations of skill acquisition during treatment. Additional research on the relationship between treatment duration and skill acquisition may serve to inform both clinicians and parents regarding potential treatment outcomes, reduce attrition, and increase parental involvement in treatment.

A limitation of this study is that treatment hours were not randomly assigned. There are a number of reasons why some participants had more treatment hours than others. These include the participant’s geographical location, the clinician’s treatment intensity recommendation, the participant’s availability, the availability of therapists to implement treatment, and the participant’s funding source. Since these data are not readily available, the assignment of treatment hours were not included as covariates in the current analysis. In addition, the number of hours spent on each domain were not differentially recorded, so the hours were distributed proportionally based upon the number of learning objectives targeted in each domain. Although this was the most objective procedure for estimating the treatment intensity for each domain, future studies should track the amount of time spent on each skill domain explicitly. Finally, data on other services that participants may have been receiving was not available and therefore were not included as a variable in this study. It is possible that some participants were receiving additional services, such as speech therapy, occupational therapy, and/or behavioral intervention through the school system. However, given that all participants received a minimum of 20 h of therapy per month, it is unlikely that participants were receiving any other concurrent intensive ABA services.

Using mastered learning objectives as the dependent variable may be seen as a strength of this study because it provides a socially significant, fine-grained analysis of treatment progress. However, it also brings with it limitations. For example, some learning objectives may be more complex than others and consequently would require more time to master than others. Further, definitions of behavioral objectives can vary across participants. However, using a standard treatment tracking system, such as Skills, can help to mitigate these limitations. Future research should be conducted to evaluate the degree to which mastery of learning objectives reflects change in more broad or global measures such as standardized assessments.

Using big data analytics to predict future learning rates based on child-specific and treatment-specific variables can provide clinicians, educators, policy makers, and parents with insight on how children with ASD will respond to ABA treatment. The current findings are among the first to evaluate treatment dosage relationships across these eight treatment domains and to identify that treatment dosage relationships are stronger for particular curricular domains than for others. In order for programs to be fully individualized for children with ASD, clinicians must consider the skill deficits of the individual and distribute treatment hours accordingly in order to yield optimal treatment results. Future research should evaluate other covariate factors in treatment dosage and learning rates. The identification of areas that respond more robustly to increased treatment intensity and duration has the potential to help inform treatment decisions, such as what percentage of therapy hours to spend on different skills. In addition, researchers should identify other child-specific factors (for example, age, gender and so on) and treatment-specific factors (for example, supervision, parent training and so on) that predict positive treatment outcomes. On the basis of these predictors, clinicians may be able to design more efficient individualized treatment programs that yield greater treatment outcomes.

## Figures and Tables

**Table 1:** Examples of target lessons and learning objectives

*Lesson*	*Domain*	*Lesson section*	*Discriminative stimulus*	*Response*	*Learning objective*
Actions	Language	Present tense tacts	The therapist presents a picture of an action or points to an action occurring in the natural environment and presents the vocal stimulus, ‘What is (pronoun/person) doing?’	‘(Action-ing)’ or ‘(Pronoun/person) is (action-ing).’	Example 1: waving Example 2: kicking
Colors	Academic	Matching/sorting	Using color cards or objects, the therapist presents a field of comparison colors, hands the child a sample color, and presents the vocal stimulus, ‘Put with same.’	The child matches the sample color to a comparison color in the field.	Example 1: green Example 2: yellow
Greetings and salutations	Social	Responding to greetings and salutations	The child is greeted.	The child makes eye contact with the greeter+(nonvocal and/or vocal response).	Example 1: eye contact+waves Example 2: eye contact+vocal, ‘hi’
Prepositions	Language	Receptive	The therapist presents a reference object, hands the child another object, and presents the vocal stimulus, ‘Put (preposition).’	The child places the object (preposition) the reference object.	Example 1: on Example 2: behind

**Table 2:** Domain specific linear regression models

*Domain*	*Intercept*	*Intensity*	*Duration*	*R*^*2*^	*p*
Academic	−26.79	1.68	4.70	0.62	<0.001
Adaptive	−6.08	0.63	1.88	0.65	<0.001
Cognitive	−14.18	1.69	2.64	0.59	<0.001
Executive	−9.58	0.83	2.46	0.60	<0.001
Language	−64.64	1.85	9.02	0.63	<0.001
Motor	−7.08	0.78	2.01	0.67	<0.001
Play	−10.12	1.06	2.25	0.58	<0.001
Social	−16.80	1.28	3.27	0.50	<0.001
